# The Pathological Department of a Modern Hospital

**Published:** 1904-04-09

**Authors:** 


### THE PATHOLOGICAL DEPARTMENT OF A MODERN HOSPITAL.

In the current number of *St. Bartholomew's Hospital Journal* a very interesting account is given by Dr. F. W. Andrewes of the pathological department. He states that St. Bartholomew's was the first metropolitan hospital to found a special pathological department. This was in 1893, when the late Dr. Kanthack was appointed lecturer on pathology on the understanding that he would devote himself solely to the subject. Since that time the growth of the department has been rapid and for some time now has been beyond the capacity of the existing buildings. The work falls under three headings:—(1) the hospital work proper—the examination of material from the wards, the operating theatres and the dead-house, with the view of diagnosis and prognosis; (2) the teaching of students; (3) original research on the material so abundantly provided in a large hospital.

The first of these entails an enormous amount of labour. Blood examinations are required in a variety of conditions, to ascertain the numbers and characters of its cellular constituents, the presence of parasites, and the presence or absence of the typhoid reaction. Suspected cases of diphtheria and tubercle give a good deal of work, while urines, fluid from cysts, and so forth, are continually being sent to the pathological department for investigation. Two or three years ago the number of tasks in clinical pathology had so increa-ed that a change was introduced. The hospital provided adequate microscopes, hæmocytometers, and other requisite apparatus for use in the wards. The junior house physicians were made to attend a special class in clinical pathology before entering upon their duties, and upon them was laid the task of instructing the clinical clerks on the subject. This has enabled the simpler examinations to be carried out in the wards, though some six or seven hundred investigations, and these the most laborious ones, are still down in the pathological department each year.

Histological and bacteriological work comes also from the operating theatres in large amount. All specimens from operations are taken to the museum where they receive critical consideration. Every specimen demanding a microscopic diagnosis is cut and stained; the sections are kept for reference, records are preserved, and the results entered in the ward notes.

Modern surgery also requires assurances as to the sterility of the hands, sponges, ligatures, and other details used in an operation, and several of the surgeons as a matter of routine have bacteriological examinations made of the skin of their own hands and that of the house-surgeon, dresser and nurse on each operating day. Sponges and ligatures also are sometimes tested as to their sterility.

The teaching staff of the Pathological Department is as follows :—In addition to the lecturer on pathology the school avails itself of the services of Dr. Klein in bacteriology and of Dr. Garrod in chemical pathology. There are two demonstrators and two junior demonstrators of pathology—one of each devoted more especially to medical and one to surgical work. Medical demonstratorships of morbid anatomy are attached to the post of Medical Registrar, and there is a demonstrator of surgical pathology, who may or may not be the Surgical Registrar. There is also a junior curator of the museum, who does a large amount of pathological work, though he has no official teaching functions; and the tutor in gynæcology and obstetrics voluntarily performs a good deal of pathological work in his own department.

What is required now is sufficient and proper accommodation, including a large, airy post-mortem room, with at least six tables, and a proper refrigerating mortuary, in which bodies can be kept without any trace of decomposition in the hottest weather. Attached to the post-mortem room there should be a small laboratory in which frozen sections could be cut, simple chemical examinations carried out, and bacteriological cultivations made. A clinical laboratory should be attached to each ward; and there should be at least three large central clinical laboratories where the more complex investigations could be carried out and provision made for all the requirements of teaching. These would be devoted respectively to chemistry, bacteriology and histology, and each should be under the control of a man specially occupied in the particular subject. In addition there should be research laboratories and a pathological library, as distinct from the general students' library. It is greatly to be hoped that, whatever difficulties may have to be surmounted, the scheme of reconstruction will provide the most perfect arrangements for the practice and teaching of pathology, the more so as St. Bartholomew's has played a leading part in bringing about general recognition of the fact that pathology is the basis of medicine and surgery. We understand that a special appeal is being made to Bart's men for subscriptions and donations towards the Pathological Block to be erected as part of the new hospital. Doubtless the patriotism of present and past students will ensure that the £15,000 required for this purpose will be speedily forthcoming.

